# Photoresponsive Metal-Organic Frameworks as Adjustable Scaffolds in Reticular Chemistry

**DOI:** 10.3390/ijms23137121

**Published:** 2022-06-27

**Authors:** Adrian Saura-Sanmartin

**Affiliations:** Departamento de Química Orgánica, Facultad de Química, Campus de Espinardo, Universidad de Murcia, E-30100 Murcia, Spain; adrian.saura@um.es

**Keywords:** metal-organic frameworks, light, photoresponsive materials, reticular chemistry

## Abstract

The easy and remote switching of light makes this stimulus an ideal candidate for a large number of applications, among which the preparation of photoresponsive materials stands out. The interest of several scientists in this area in order to achieve improved functionalities has increase parallel to the growth of the structural complexity of these materials. Thus, metal-organic frameworks (MOFs) turned out to be ideal scaffolds for light-responsive ligands. This review is focused on the integration of photoresponsive organic ligands inside MOF crystalline arrays to prepare enhanced functional materials. Besides the summary of the preparation, properties and applications of these materials, an overview of the future outlook of this research area is provided.

## 1. Introduction

Metal-organic frameworks (MOFs) are a type of crystalline porous material having organic ligands connected to metal clusters [1,2,3,4,5,6]. This type of material shows a terrific design adaptability due to the almost unlimited combinations of metallic salts and organic ligands [7,8]. Several scientists have been attracted by this important part of reticular chemistry [9,10,11,12], probably due to the fact that the researcher’s inventiveness is the only limitation in this research field. Thus, different research groups have greatly contributed to the exponential growth of this area, using these materials in a diverse range of applications, including catalysis [13,14,15,16,17,18,19], water harvesting [20,21,22,23], biomedicine [24,25,26] and sensing [27,28,29,30].

The development of organic ligands having a higher structural complexity is one of the main directions in order to prepare MOFs showing advanced functionalities [31,32,33,34]. Thus, the incorporation of stimuli-responsive scaffolds inside a MOF matrix turned out to be an ideal strategy within reticular chemistry [35,36,37,38], allowing to prepare smart materials [39,40]. The application of an external stimulus induces changes in the organic ligands, leading to modifications of certain properties of the metal-organic material, such as porosity [41,42]. The selected stimulus should be able to promote modifications in the crystalline array without causing damage to its structural integrity.

Among the available stimuli, light is particularly appealing due to: (i) the easy and remote switching control; (ii) the possibility of varying the intensity, wavelength and irradiation time; (iii) the clean and non-destructive operation [43]. Different organic ligands bearing photoactive units have been employed for the preparation of photoresponsive MOFs [44,45,46].

Different strategies can be carried out for this purpose, involving both the design of the photoresponsive ligands and the incorporation of the photoresponsive units by postsynthetic modifications (PSM) [1,2]. The direct assembly of the photoactive ligands directly affords the target material, but the ligand should retain its structural integrity during the MOF formation reaction. In stark contrast, PSM strategies lead to the preparation of materials containing the desired units by using a premade crystalline framework. Thus, ligands that are not stable to the conditions of formation of the metal-organic material can be used in order to achieve a greater variety of functionalization. However, there are two main limitations: (i) the postsynthetic conditions must not affect the integrity of the crystalline array; (ii) the functionalization with the photoresponsive units may not be homogeneous throughout the material.

**Figure 1:**
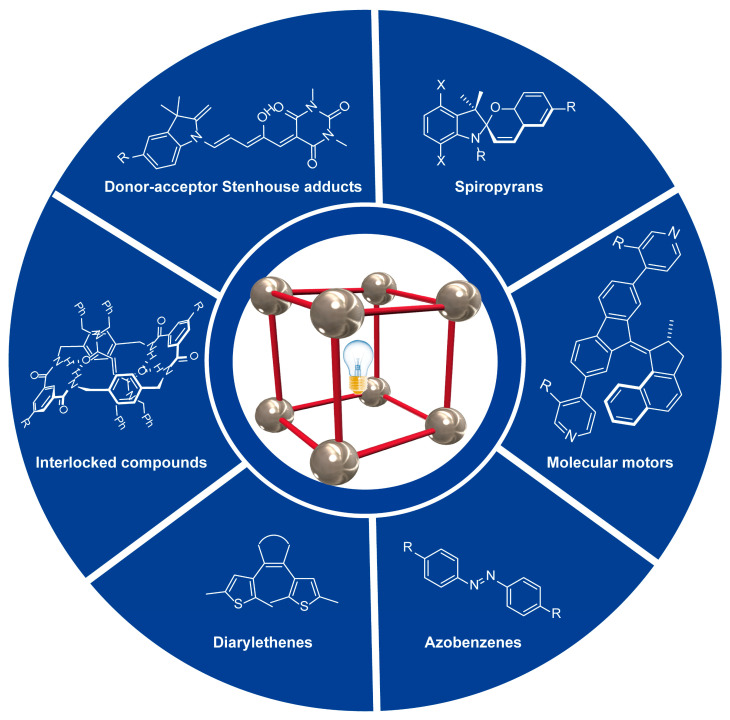
Selected photoactive organic ligands employed to prepare light-responsive metal-organic frameworks.

This short review aims to give an insight in the preparation and application of light-responsive metal-organic frameworks by using different photoswitchable organic ligands (Figure 1), including azobenzenes [47], diarylethenes [48], spiropyrans [49], donor-acceptor Stenhouse adducts (DASA) [50], molecular motors [51], interlocked fumaramides [52] and interlocked styrylpyridinium derivatives [53]. For this purpose, some recent advances in the use of the abovementioned organic ligands will be highlighted in this manuscript.

## 2. Azobenzene-Based MOFs

**Figure 2:**
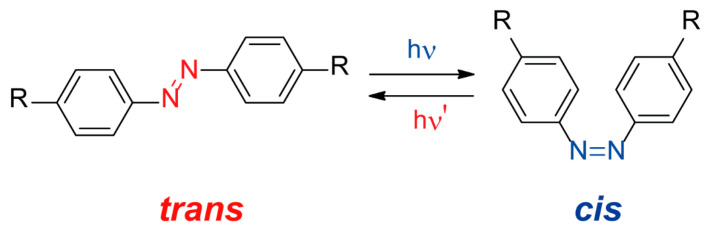
Photoisomerization of an azobenzene compound showing both geometric isomers, *trans* (shown in red) and *cis* (shown in blue).

Azobenzenes are a type of organic compound constituted by two benzene rings connected through an azo functionality. The huge variations in the shape, polarity and size induced by *cis*/*trans* photoisomerization (Figure 2) makes them one of the most extensively studied photoswitchable molecules [54,55,56,57]. Azobenzenes are postulated as convenient scaffolds to induce a photoresponsive behavior in condensed materials through easy isomerization between both geometric isomers [58]. The employment of azobenzene-based compounds as organic ligands in the preparation of MOFs led to different applications such as control over the pore (open/close pore) [59] or capture and release of carbon dioxide [60,61].

**Figure 3:**
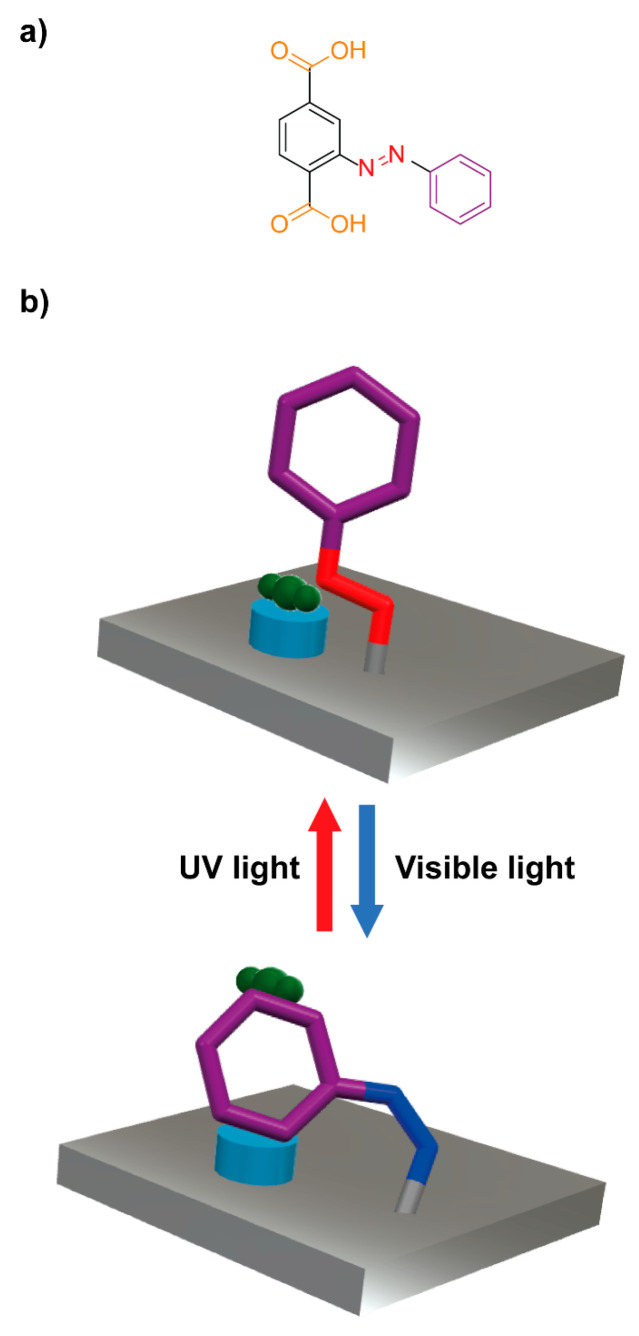
(**a**) Chemical structure of the azobenzene ligand **1**; (**b**) cartoon representation of the operation of the azobenzene-functionalized UiO-66 composite as efficient adsorbent for tailorable CO_2_ adsorption [62]. Color key: metallic grey = azobenzene-functionalized UiO-66 (**U-azo**); red = nitrogen atoms of the trans isomer; dark blue = nitrogen atoms of the cis isomer; purple = phenyl ring; dark green = CO_2_ molecule; white = hydrogen atoms; light blue = TEPA site.

Professors Liu, Sun and colleagues prepared azobenzene-functionalized UiO-66 absorbents [62]. First, the reaction of 2-phenyldiazenyl terephthalic acid (**1**) (Figure 3a), zirconium tetrachloride and acetic acid under solvothermal conditions afforded the crystalline material **U-azo** having azobenzene ligands connected to Zr_6_O_4_(OH)_4_ clusters through the carboxylate groups. This porous material was suspended in a solution of tetraethylenepentamine (TEPA) in anhydrous methanol and stirred under inert atmosphere for 12 h. After removing the solvent, MOF-based composite materials having up to 10.8 wt% of TEPA were obtained. The TEPA sites embedded in the material allow a tailorable CO_2_ capture, establishing strong interaction with this guest gas when the *trans* isomer of the azobenzene is connecting the different metal clusters (Figure 3b, above). Upon visible irradiation of the MOF surface, the *trans* isomer was photoisomerized to the *cis* azobenzene, thus positioning the aromatic ring on the TEPA sites (Figure 3b, below), reducing the CO_2_ adsorption capacity of the composite. Ultraviolet irradiation of the isomerized material led to the *trans* isomers, showing a remarkable modification of the adsorbed CO_2_ amount up to 45.6% through cyclic irradiation.

**Figure 4:**
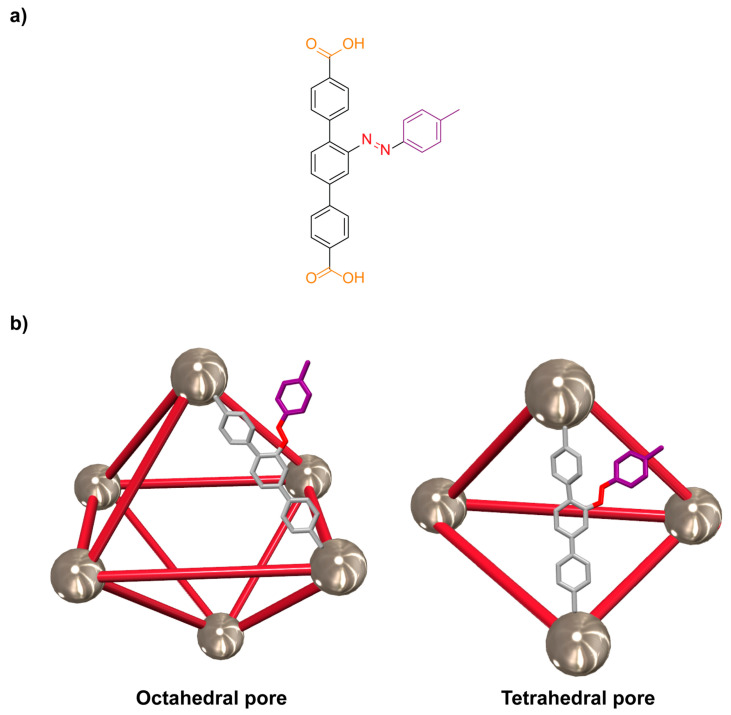
(**a**) Chemical structure of the azobenzene ligand **2**; (**b**) cartoon representation of the octahedral and tetrahedral pores of the azobenzene-functionalized thorium organic framework **Th-Azo-MOF** [63]. For clarity, only the structure of one organic ligand per pore is shown. Color key: metallic grey = thorium clusters; red = rods representing the azobenzene ligand **2** and nitrogen atoms of the azo functionality; light grey = carbon atoms; white = hydrogen atoms; purple = phenyl ring.

Professors Mei and coworkers described the preparation of the thorium-organic framework **Th-Azo-MOF** through the reaction of the azobenzene-modified photoswitchable ligand **2** (Figure 4a) and thorium nitrate hexahydrate in the presence of small amounts of nitric acid and water, using *N,N*-dimethylformamide (DMF) as solvent [63]. This three-dimensional material, having an overall formula [Th_6_O_4_(OH)_4_(H_2_O)_6_(**2**)_6_], formed huge tetrahedral and octahedral pores (Figure 4b) with an impressive 73.7% of solvent-accessible void volume. The authors monitored the dynamics of this porous material related to *cis/trans* photoisomerization by UV-vis absorption and ^1^H NMR. Irradiation at 365 nm afforded a material having 19.7% of *cis* azobenzene ligands after 120 min. The reversible isomerization is also possible by using blue light (460 nm) or heating at 60 °C under dark conditions. The irradiation experiment only decreased the amount of *cis* ligand to 5.51%, while the heating treatment allows the recovery of the pristine material. These actinide MOFs were applied as efficient adsorbents of Rhodamine B, showing an adsorption capacity of 100.01 (±0.68) mg/g in the pristine material. This capacity was slightly reduced to 97.9 (±0.61) mg/g after 1 h of irradiation, showing the potential of these materials in smart photo-induced release applications.

## 3. Diarylethene-Based MOFs

**Figure 5:**
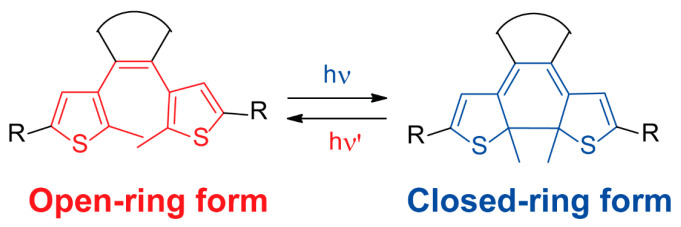
Photoisomerization of a diarylethene compound showing the open ring (shown in red) and closed ring (shown in blue) forms.

Diarylethenes are a type of light-responsive organic compound constituted by aromatic rings connected through a carbon–carbon double bond [64]. These molecules easily exchange the open ring and closed ring forms by the application of a photochemical stimulus (Figure 5). Along with their diverse structural functionalization, diarylethenes are suitable candidates for the development of photoresponsive metal-organic porous materials due to their exceptional photoreactivity and thermal stability [65,66,67].

**Figure 6:**
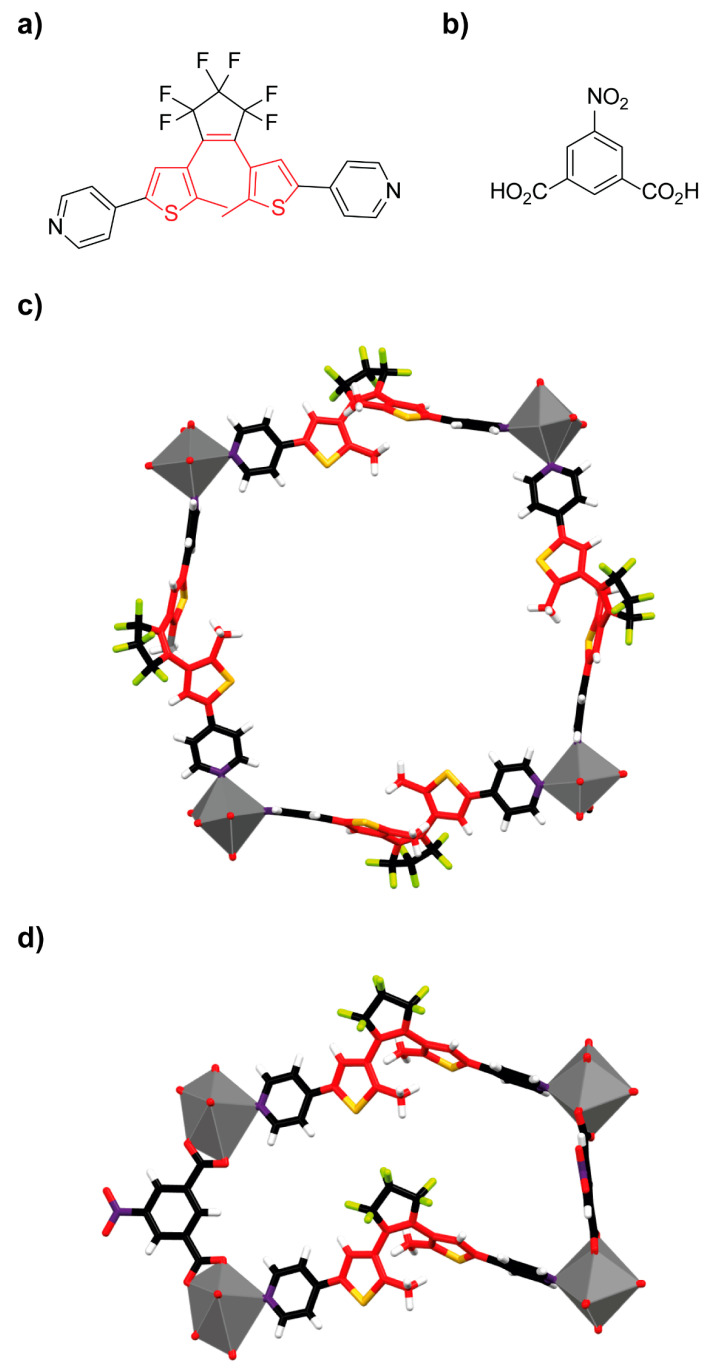
(**a**) Chemical structure of the diarylethene linker **3**; (**b**) structure of **nip^2−^**; (**c**) stick representation of the crystal structure showing the square-shaped macrocycles structure of the diarylethene-based **^DTE^MOF**; (**d**) stick representation of the crystal structure of **^DTE^MOF** showing the connections between one side of two different macrocycle-shaped diarylethene-containing metallogrids [68]. Interpenetration is omitted for clarity in the solid structure. Color key: grey = cadmium atoms; black = carbon atoms; red = oxygen atoms and carbon atoms of the diarylethene motif; white = hydrogen atoms; yellow = sulfur atoms; green = fluor atoms; purple = nitrogen atoms.

Professors Sato, Aida and colleagues reported the synthesis of the cadmium-organic framework **^DTE^MOF** constituted by the bipyridine-functionalized diarylethene ligand **3** (Figure 6a) and 5-nitroisophthalate (**nip^2−^**) (Figure 6b) [68]. The reaction of the organic linker **3**, **nip^2−^** and cadmium nitrate using a mixture of DMF and methanol as solvent afforded colorless prismatic crystals of the target MOF. The single crystal structure revealed that this material crystallizes in the *P4_2_/n* space group of the tetragonal system, in which Cd^2+^ adopts a pentagonal bipyramidal geometry. The pentagonal plane is occupied by two **nip^2−^** carboxylates and a nitrogen atom from one of the bipyridyl moieties of the diarylethene ligand **3**, while the axial positions are coordinated to other bipyridyl group of the ligand **3** and to a water molecule. The solid structure showed four cadmium atoms connected by four diarylethene ligands forming a square-shaped macrocycle (Figure 6c), where the square grids composed of cadmium and ligand **3** are connected by **nip^2−^** along the *c*-axis (Figure 6d) affording a nanotubular structure. These metallogrids are mechanically interlocked with four adjacent nanotubular structures.

The carbon atoms in the thienyl groups of the diarylethene ligands are placed 3.52 Å spaced out from each other showing an antiparallel conformation, which is adequate for carrying out a photocyclization reaction [69]. Thus, the irradiation of a suspension of **^DTE^MOF** in DMF/MeOH at 305–315 nm instantly turned the colorless crystals of the metal-organic porous material into a homogeneous dark blue solution. The photostationary state was reached after 2 h, leading to a mixture having 80% of closed ring diarylethene linkers. This photocyclization reaction led to the breaking of the coordination bonds between carboxylate and pyridyl groups and cadmium atoms, degrading the crystalline array. Further attempts to obtain a metal-organic framework using the closed form of **3** as starting material were unsuccessful, suggesting that the geometrically demanding closed ring form could not be fitted to a coordination geometry in order to make the target MOF. Interestingly, the reversible isomerization can photochemically regenerate the initial **^DTE^MOF** by exposing a solution with the obtained fragments to visible light. This smart photochemically breakable and recoverable metal-organic material paves the way to the development of enhanced applications in greatly effective switchable release of guest compounds encapsulated within a MOF matrix.

**Figure 7:**
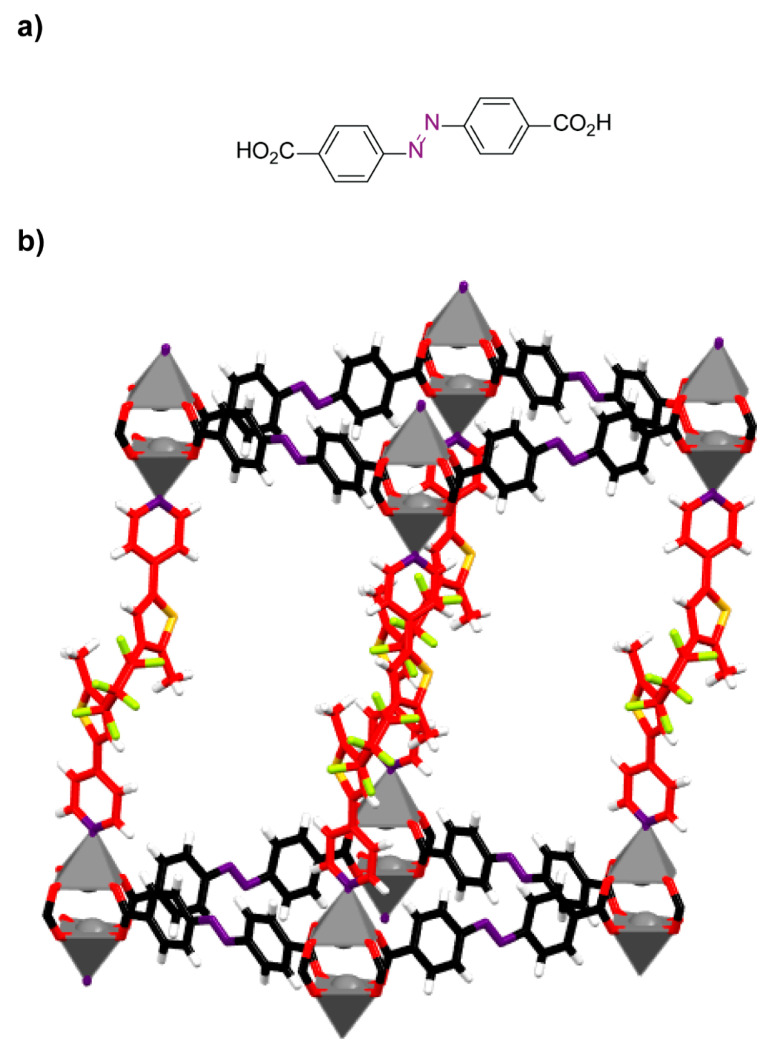
(**a**) Chemical structure of azobenzene **4**; (**b**) stick representation of the crystal structure **of ECUT-30** showing a portion of the lattice framework [70]. Interpenetration is omitted for clarity in the solid structure. Color key: grey = zinc atoms; black = carbon atoms of the azobenzene ligand **4**; red = oxygen atoms and carbon atoms of the diarylethene ligand **3**; white = hydrogen atoms; yellow = sulfur atoms; green = fluor atoms; purple = nitrogen atoms.

Professors Zheng, Luo and coworkers prepared the zinc-organic framework **ECUT-30** combining two photoresponsive organic ligands through the solvothermal reaction of zinc nitrate, the dipyridine-functionalized diarylethene **3** (Figure 6a) and the azobenzene-based ligand **4** (Figure 7a) bearing two carboxylate groups [70]. The solid structure of the synthesized metal-organic porous material (Figure 7b) revealed that the MOF crystallizes in the monoclinic *C2/c* space group, with a five-coordinated pyramidal zinc(II) site having attached four azobenzene oxygen atoms and one pyridinic nitrogen atom of the diarylethene ligand **3**. The zinc paddlewheel clusters are connected by four carboxylate groups of azobenzene ligands **4**, creating a four-fold interpenetration. This material was employed in the capture of C_2_H_2_, C_2_H_4_ and CO_2_, obtaining a fine-tuning adsorption selectivity towards different guests by the application of a photochemical stimulus.

## 4. Spiropyran-Based MOFs

**Figure 8:**
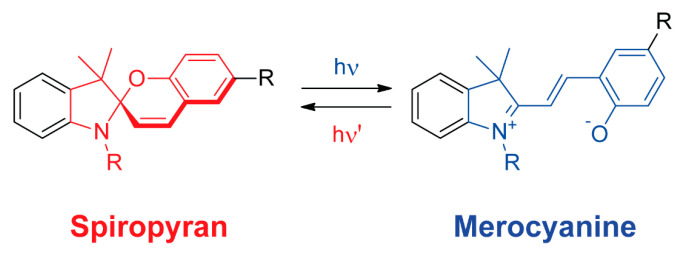
Reversible photoisomerization of a spiropyran compound (shown in red) to a merocyanine derivative (shown in blue).

Spiropyrans are a type of organic compounds in which a pyran ring is bonded to a second ring, usually a heterocyclic compound, in a spiro way [71]. These molecules are widely known for their photochromic properties, reversibly changing to merocyanines by photochemical irradiation (Figure 8). The employment of spiropyrans ligands in the construction of metal-organic frameworks also led to photoresponsive crystalline porous materials that can be employed in different applications, such as photophysics modulation [72,73] and light-dependent gas adsorption [74]. 

**Figure 9:**
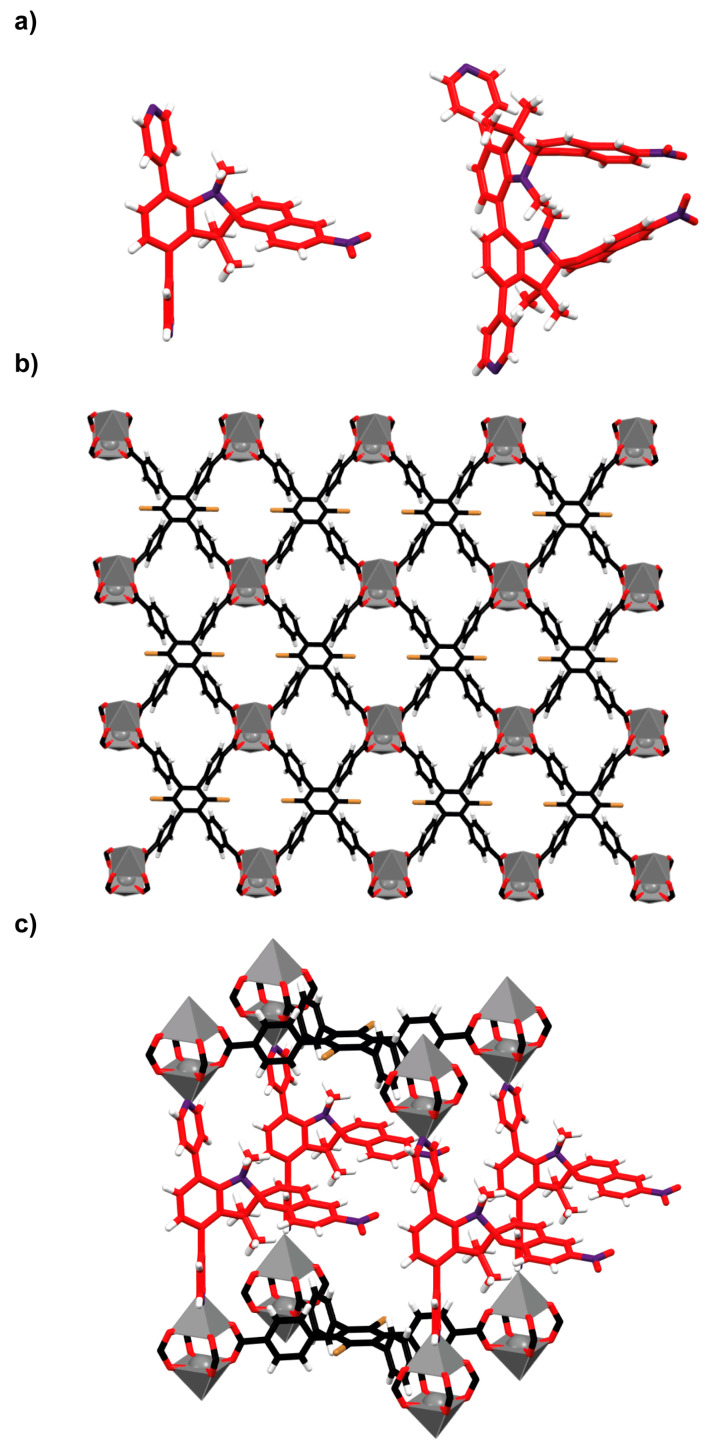
(**a**) Stick representation of the crystal structures of spiropyran-based organic ligands **5** (**left**) and **6** (**right**); (**b**) stick representation of the crystal structure of the two-dimensional premade **^DBTD^MOF**; (**c**) simulated representation of the superposition of crystal structures of ligand **5** and **^DBTD^MOF** showing a three-dimensional arrangement (**^Spiro−1^MOF**) [75]. Color key: grey = zinc atoms; black = carbon atoms of the ligand **7**; red = oxygen atoms and carbon atoms of the spiropyran ligands **5** and **6**; white = hydrogen atoms; yellow = bromine atoms; purple = nitrogen atoms.

Professor Shustova and collaborators reported the control over cycloreversion kinetics using spiropyran ligand-based zinc-organic frameworks [75]. With this aim, the researchers carried out a synthetic approach in which the spiropyran ligand (**5** or **6**) bearing pyridyl moieties (Figure 9a) is attached to zinc(II) paddlewheel clusters of the premade **^DBTD^MOF**, constructed with a tetracarboxylate organic linker **7** (Figure 9b), thus connecting different 2D layers of this premade MOF matrix to afford a three-dimensional structure (Figure 9c).

The authors studied the photochromic cycloreversion kinetics of ligand **5** both in solution and within the crystalline array of **^Spiro−1^MOF**, demonstrating that the material mimics the solution behavior. Thus, a MOF matrix affords a photoswitchable isomerization in the solid state providing an arrangement having enough void space in comparison to the crowded packing observed for the free ligands. However, **^Spiro−2^MOF**, in which the organic ligand **6** bearing two photoswitchable spiropyran groups was used, exhibited a limited photoisomerization. These results can be explained by the confined environment that provides a high steric hindrance to the spiropyran linkers.

Additionally, **^Spiro−1^MOF**, in which spiropyran **5** connects different layers, was employed to map the acidic degradation of the material by using photoluminescence spectroscopy and powder X-ray diffraction (PXRD). This work showed the fine-tuning of the photoisomerization rate of spiropyrans inside MOFs as a function of the structure of the framework, and, also, provided a toolbox for the development of a new class of light-responsive markers.

The same research group also studied the tailoring and tunability of the optoelectronic properties of mono- and heterometallic actinide-containing spiropyran-based metal-organic frameworks [76]. This work demonstrated that the electronic properties of these materials can be tuned by different approaches: (i) incorporation of photoswitches; (ii) integration of a secondary metal; (iii) inclusion of a guest inside the MOF pores. This research group went a step further and developed the first photochromic field-effect transistor (FET) based on a zirconium-organic framework backbone encapsulating 7,7,8,8-tetracyanoquinodimethane, along with its incorporation into a two-light-emitting diode (LED) failsafe circuit. The drain current of the FET system could be regulated by the application of different gate voltages, and, also, ultraviolet irradiation that induces the conversion of the spiropyrans ligands to the merocyanine linkers.

## 5. DASA-Based MOFs

**Figure 10:**
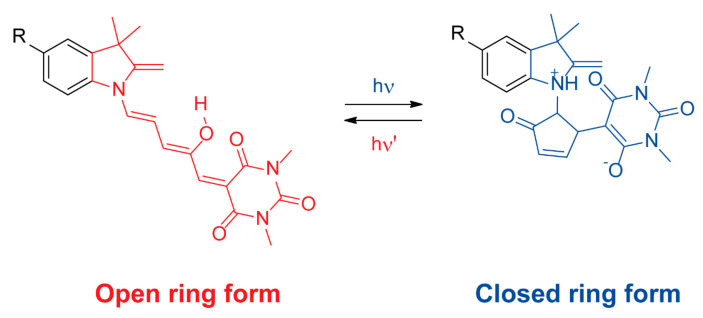
Reversible photoisomerization of a donor-acceptor Stenhouse adduct: from its open ring form (shown in red) to its closed ring form (shown in blue).

Donor-acceptor Stenhouse adducts are a type of heterocyclic photoswitchable organic compound that experiences reversible triene cyclisation through the application of a photochemical stimulus (Figure 10), efficiently exchanging between open ring and closed ring forms [77].

**Figure 11:**
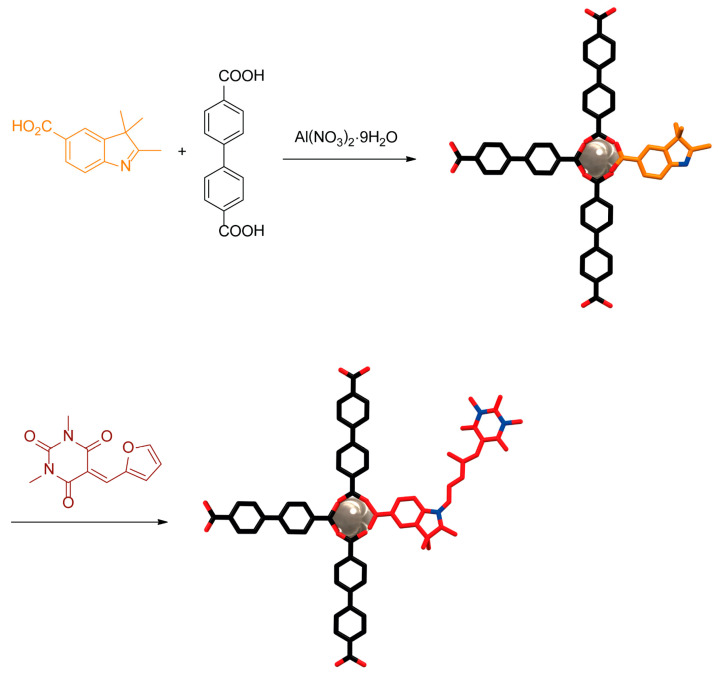
Schematic representation of the mixed linker solvothermal protocol to obtain the defect engineered **DUT-5(indoline)_0.5–2.5_** (above) and postsynthetic modification to prepare the DASA **DUT-5(indoline)_0.5–2.5_(DASA)** (below) [78]. Color key: grey = aluminum atom; black = carbon atoms of the dicarboxylic acid **9**; orange = carbon atoms of the indoline **8**; red = oxygen atoms and carbon atoms of the DASA ligand; white = hydrogen atoms; blue = nitrogen atoms.

Professor D’Alessandro and colleagues reported the postsynthetic modification of defect engineered MOFs to obtain DASA-functionalized crystalline porous materials [78]. The preparation of the defect engineered **DUT-5(indoline)_0.5–2.5_** was carried out by a mixed-linker solvothermal reaction where the indoline **8** bearing a carboxylate motif and biphenyl-4,4′-dicarboxylic acid (**9**) reacts with aluminum nitrate. Thus, defective indoline sites are introduced in the crystalline array. The subsequent reaction of these indoline sites with 5-(furan-2-ylmethylene)-1,3-dimethylpyrimide (**11**) afforded the **DUT-5(indoline)_0.5–2.5_(DASA)** (Figure 11).

This porous material exhibited a bistable behavior, which the closed ring form, obtained upon white light irradiation, turned out to be stable for up to 30 days. After a heating treatment, the material underwent the regeneration of the open ring form of the DASA ligands. Furthermore, the stability of the system was tested under multiple photoswitching-heating cycles, observing a partial deterioration of the crystalline matrix. Despite the short-term durability of this system, its bistability could serve for applications in which an extended permanence of both bistable forms is a requirement, such as the development of non-volatile memory (NVM) materials.

## 6. Molecular Motor-Based MOFs

A synthetic molecular motor is a molecular machine-type compound that experiences a rotational motion upon activation [79,80].

**Figure 12:**
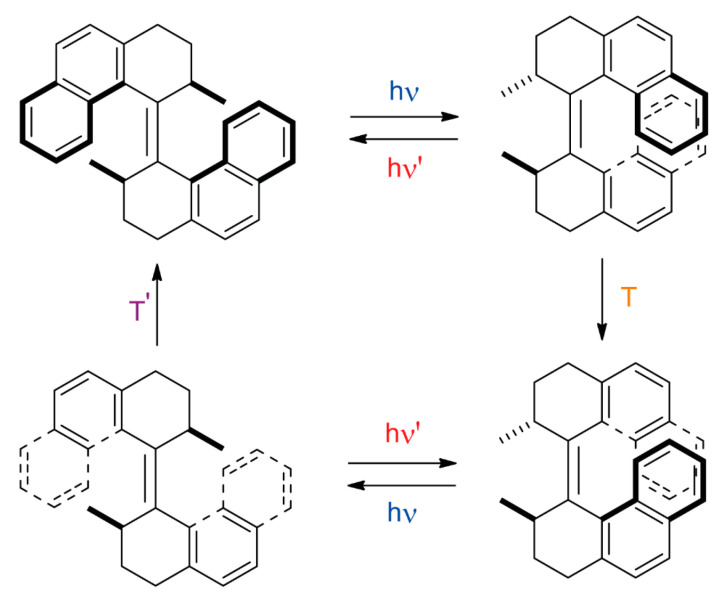
Four-step isomerization cycle of the photoresponsive overcrowded alkene-based molecular motor **12** prepared by Professor Feringa’s research group [83].

One of the topics of the Nobel Prize in Chemistry in 2016 was the development of molecular motors envisioned by Professor Feringa [81]. The Nobel Prize laureate researcher has substantial expertise in the preparation of light-driven unidirectional rotary molecular motors [82,83]. In 1999, his research group reported the preparation and operation of the overcrowded alkene-based molecular motor **12** [84], in which a unidirectional rotary motion was accomplished by a four-step isomerization cycle (Figure 12).

The development of enhanced molecular motors makes this type of molecular machine a tunable scaffold in several fields of research, including biomolecular technology and smart materials [51].

The well-defined spatial organization provided by the MOF matrix led to the precise positioning of the molecular motor linkers, overcoming the Brownian motion that precludes cooperativity in solution [51].

**Figure 13:**
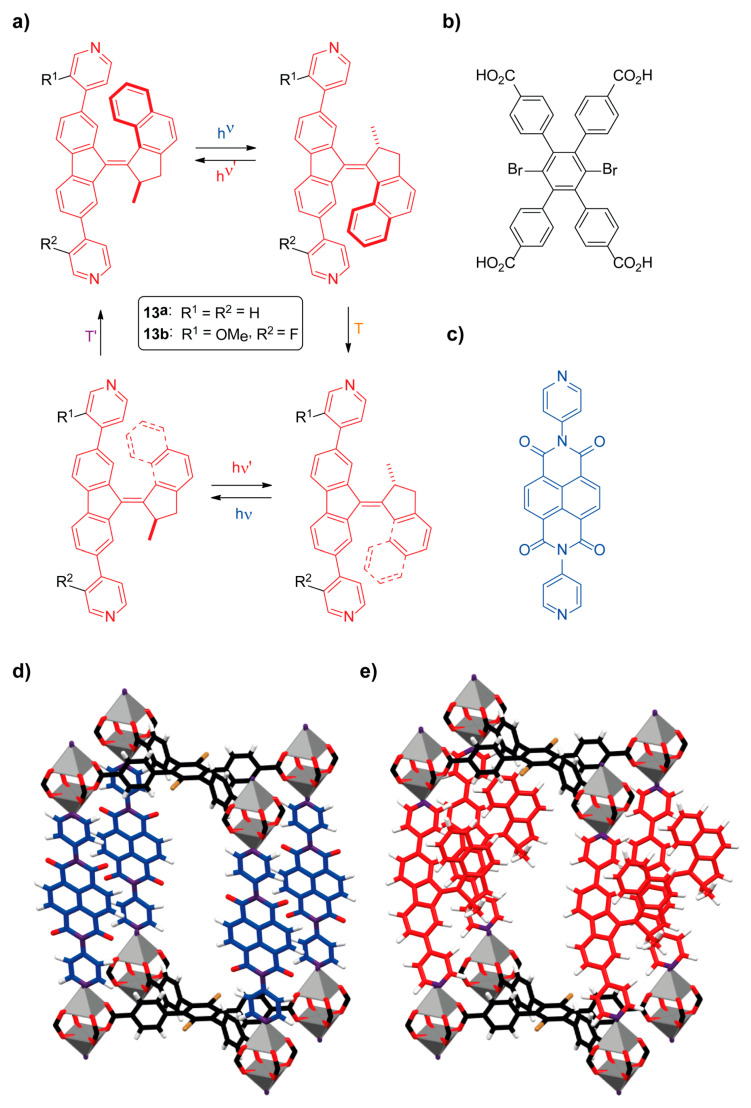
(**a**) Four-step isomerization cycle of the molecular motors **13** [85]; (**b**) chemical structure of **TCPB**; (**c**) structure of **DPNI**; (**d**) stick representation of the crystal structure of the **BrYO-MOF** showing a portion of the lattice framework [86]; (**e**) simulated stick schematic representation of the **moto-MOF1** containing the overcrowded alkene-based molecular rotor **13a** as organic linker [85]. Color key: grey = zinc atoms; black = **TCPB** carbon atoms; blue = **DPNI** carbon atoms; red = oxygen atoms and carbon atoms of the overcrowded alkene-based molecular motor linker **13a**; white = hydrogen atoms; brown = bromine atoms; purple = nitrogen atoms.

Professors Browne, Wezenberg, Feringa and coworkers reported the first preparation of a zin-organic framework containing overcrowded alkene-type molecular motors as linkers [85]. The molecular motor-based linkers **13** bearing pyridyl units at the ends could experience a four-step isomerization cycle by the application of light and thermal stimuli (Figure 13a). The researchers carried out a solvent-assisted linker exchange (SALE) of the previously reported **BrYO-MOF** constituted by 1,4-dibromo-2,3,5,6-tetrakis(4-carboxyphenyl)benzene (**TPCB**) and dipyridyl-naphthalenediimide (**DPNI**) linkers (Figure 13b–d) [86]. This postsynthetic functionalization of a MOF backbone involved the exchange of the pyridine-based pillars **DPNI** with the corresponding overcrowded alkene-type motor linker **13** by immersing crystals of **BrYO-MOF** in a solution of **13** in DMF at 60 °C. Yellow crystals of **moto-MOF1** were obtained by this methodology using the organic linker **13a** (Figure 13a) after 24 h of heating. **Moto-MOF2-*E*** and **moto-MOF2-*Z*** required an additional 72 h to completely replaced the **DPNI** pillars with the molecular motor ligands **13b** (Figure 13a). The successful ligand exchange was confirmed by Raman spectroscopy, not observing bands attributable to **DPNI**.

The authors studied the photochemical and thermal isomerization of the molecular motor **13** in solution and integrated in the **moto-MOFs** matrix by using Raman spectroscopy. These results revealed that the overcrowded alkene motor struts could perform an unimpeded 360° unidirectional rotary motion in the solid state, having a similar dynamic behavior to that observed in solution. These finding are remarkably promising in the research area of molecular machinery, paving the way for the development of applications where a switchable unidirectional motion is a requirement, such as photoswitchable microfluidic pumps and photodriven mass transport.

**Figure 14:**
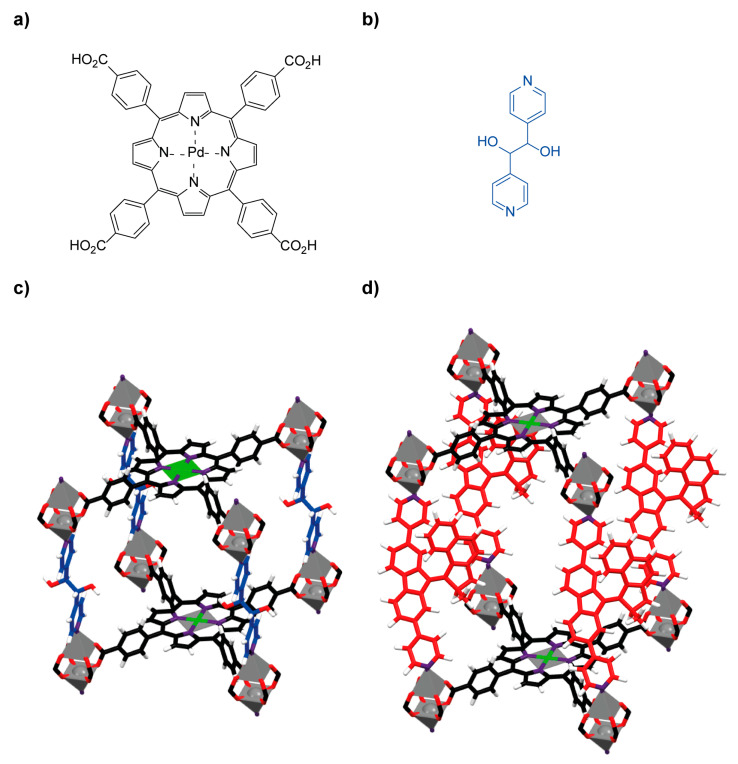
(**a**) Chemical structure of porphyrin-based photosensitizer strut **14**; (**b**) structure of **DPG** pillars; (**c**) stick representation of the crystal structure of the **PdTCPP-MOF** showing a portion of the lattice framework; (**d**) simulated stick schematic representation of the **moto-MOF3** containing the overcrowded alkene-based molecular rotor **13a** as organic linker [87]. Color key: grey = zinc atoms; grey with green lines = palladium atoms; black = carbon atoms of the photosensitizer ligand **14**; blue = **DPG** carbon atoms; red = oxygen atoms and carbon atoms of the overcrowded alkene-based molecular motor linker **13a**; white = hydrogen atoms; purple = nitrogen atoms.

The light-driven molecular motor linker **13a** was employed in a SALE functionalization protocol in the premade **PdTCPP-MOF**, constituted by *meso-α,β*-di(4-pyridil)glycol pillars (**DPG**) and the photosensitizer porphyrin-based struts **14** connected through zinc paddlewheel clusters (Figure 14a–c) [87]. The newly prepared molecular motor-type **moto-MOF3** (Figure 14d) also exhibited an unhindered rotary motion of the overcrowded alkene within the crystalline array. Interestingly, an efficient energy transfer between the **DPG** ligands and the molecular motor-based pillars **13a** was determined, allowing the photoisomerization to take place using green light as irradiation source.

## 7. Interlocked Fumaramide-Based MOFs

Mechanically interlocked molecules (MIMs) are a type of compound in which at least two components are intertwined with each other [88,89]. Along with molecular motors, the use of MIMs for the development of molecular machines was the topic of the Nobel Prize in Chemistry in 2016 [90,91,92]. Rotaxanes, a type of MIMs having at least a linear component threaded into a cyclic one, stand out for the greatest variety of motions, being possible to exert control over the dynamics of the counterparts [93,94].

**Figure 15:**
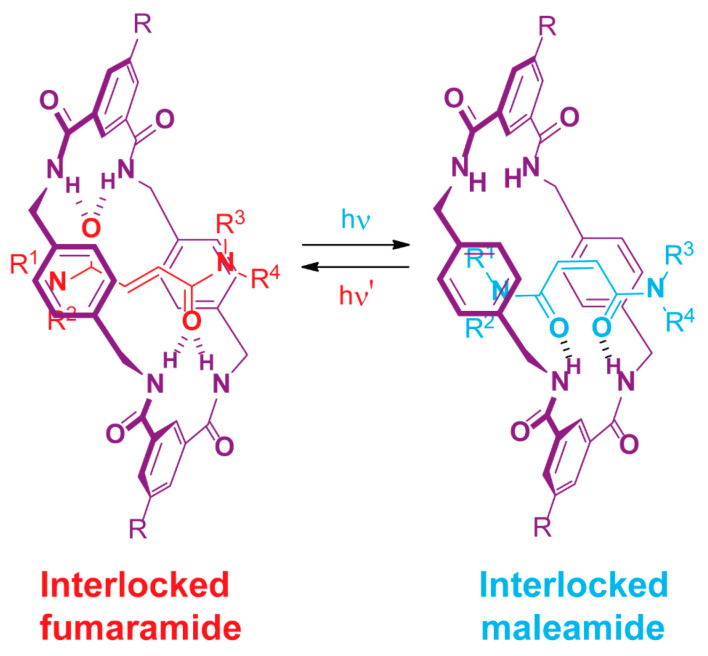
Reversible photoisomerization of an interlocked dicarboxamide, showing the fumaramide (in red) and maleamide (in blue) isomers.

Light has been shown to be an effective stimulus in rotaxanes bearing photoresponsive scaffolds [95,96,97,98]. Thus, interlocked fumaramides are postulated as a useful tool to obtain molecular machines operating through photoisomerization to their equivalent intertwined maleamides (Figure 15) [99,100,101,102].

The incorporation of rotaxanes inside a MOF matrix has turned out to be a suitable strategy to exploit the properties of this type of MIMs in the solid state [103,104,105,106], including the study of the component’s dynamics [107,108,109,110].

**Figure 16:**
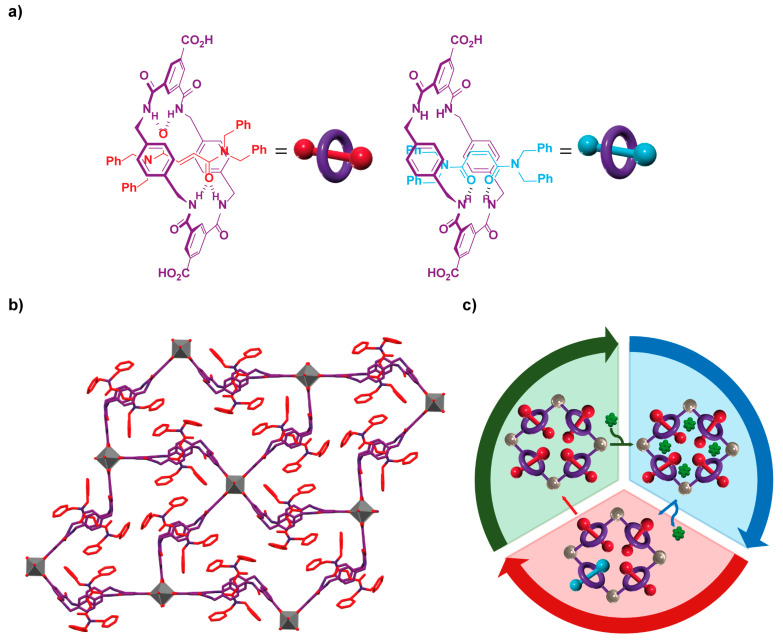
(**a**) Chemical structure of ligands **(*E*)-15** and **(*Z*)-15**; (**b**) stick representation of the crystal structure of the two-dimensional **UMUMOF-(*E*)-3** showing rhombohedral grids; (**c**) cartoon representation of the cyclic operation of **UMUMOF-(*E*)-3** as molecular dispenser, involving quinone loading, photorelease and thermal treatment [113]. Color key: grey = copper atoms; purple = carbon atoms of the benzylic amide macrocycle and nitrogen atoms; red = carbon atoms of the fumaramide and oxygen atoms; green = *p*-benzoquinone molecules; purple toroid = benzylic amide macrocycle; red rod = fumaramide thread; blue rod = maleamide thread.

As an eldest member of the MOF family having benzylic amide macrocycle-based organic ligands [111,112], Professor Berna and coworkers reported the preparation of the **UMUMOF-(*E*)-3** having the interlocked fumaramide **(*E*)-15** (Figure 16a) as an organic ligand [113]. The reaction of **(*E*)-15** and copper nitrate in the presence of nitric acid under solvothermal conditions afforded blue prismatic crystals of a MOF having an overall formula [Cu_2_(**(*E*)-15**)_2_(H_2_O)_2_(DMF)_2_]. This porous material crystallized in the monoclinic *P2_1_/n* space group, forming a two-dimensional net of non-interpenetrated rhombohedral grids, in which the copper(II) paddlewheels are coordinated to **(*E*)-15** ligands at the vertex and to water molecules at axial positions (Figure 16b). The stacking of these rhombohedral metallogrids stablished well-ordered channels along the *a*-axis. Interestingly, the material has enough free volume to undergo the fumaramide/maleamide isomerization. Thus, upon irradiation at 312 nm of a suspension of **UMUMOF-(*E*)-3** crystals in dichloromethane for 8 h, a new material having 20% of interlocked maleamides **(*Z*)-15** (Figure 16a) was obtained, showing a higher pore size compared to that of the pristine material. The authors also prepared the metal-organic crystalline material **UMUMOF-(*Z*)-3** using the interlocked maleamide **(*Z*)-15** as struts, displaying even more porosity.

The researchers envisioned the employment of these materials as molecular dispensers as the result of a breathing-like process of the pores induced by a photochemical stimulus. In order to test this idea, *p*-benzoquinone was selected as a model cargo, accomplishing its loading inside **UMUMOF-(*E*)-3** through the suspension of this material in a 1.2 M solution of quinone in chloroform for 8 h. The same isomerization conditions abovementioned, which provided 20% of maleamides struts within the crystalline array, were carried out, achieving the complete release of the cargo as a consequence of this photoirradiation. In contrast, a similar experiment with **UMUMOF-(*Z*)-3** did not require light activation to reach similar results in a shorter time. Conveniently, the photoisomerized material can be regenerated by a thermal treatment, being possible to reuse the **UMUMOF-(*E*)-3** in the operation cycle involving quinone loading, photorelease and thermal recovery. This cyclic reusability of the material showed a retaining of the cargo capacity close to 80% after three iterations.

This new approach to incorporate photoswitchable rotaxane-based components into MOFs paves the way for the development of new functional rotaxane-based materials operating as molecular machines in the solid state.

## 8. Interlocked Styrylpyridinium Derivatives

Cucurbiturils [114], a type of macrocyclic compound made of glycoluril monomers connected through methylene groups, are highly employed in supramolecular chemistry. The formation of different supramolecular complexes constituted by these macrocycles and pyridinium-based compounds had been greatly studied [115,116,117,118]. Cucurbituril-based scaffolds have been employed in the preparation of different interlocked molecules, such as pseudorotaxanes and rotaxanes [119,120,121,122,123,124]. Interestingly, some examples of interlocked cucurbituril ligands incorporated in the crystalline array of metal-organic frameworks have been reported [125,126,127,128].

**Figure 17:**
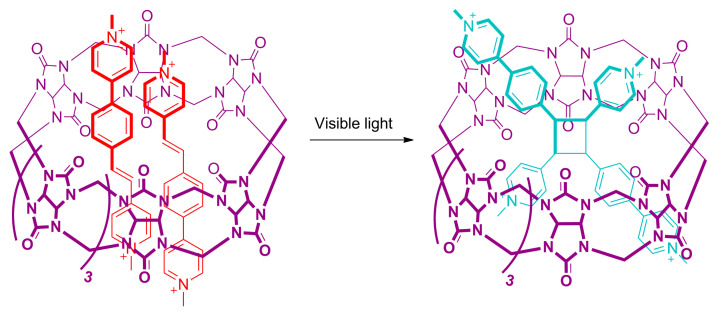
Cucurbit [8]uril-promoted [2 + 2] photodimerization reaction of styrylpyridinium guest molecules reported by Professor Gao’s research group [131].

The incorporation of photoactive guests, such as styrylpyridinium derivatives, in the cavities of cucurbituril with a macrocycle-photoactive unit binding ratio of 1:2 has led to a cucurbituril-promoted photodimerization upon visible light irradiation (Figure 17) [129,130,131].

**Figure 18:**
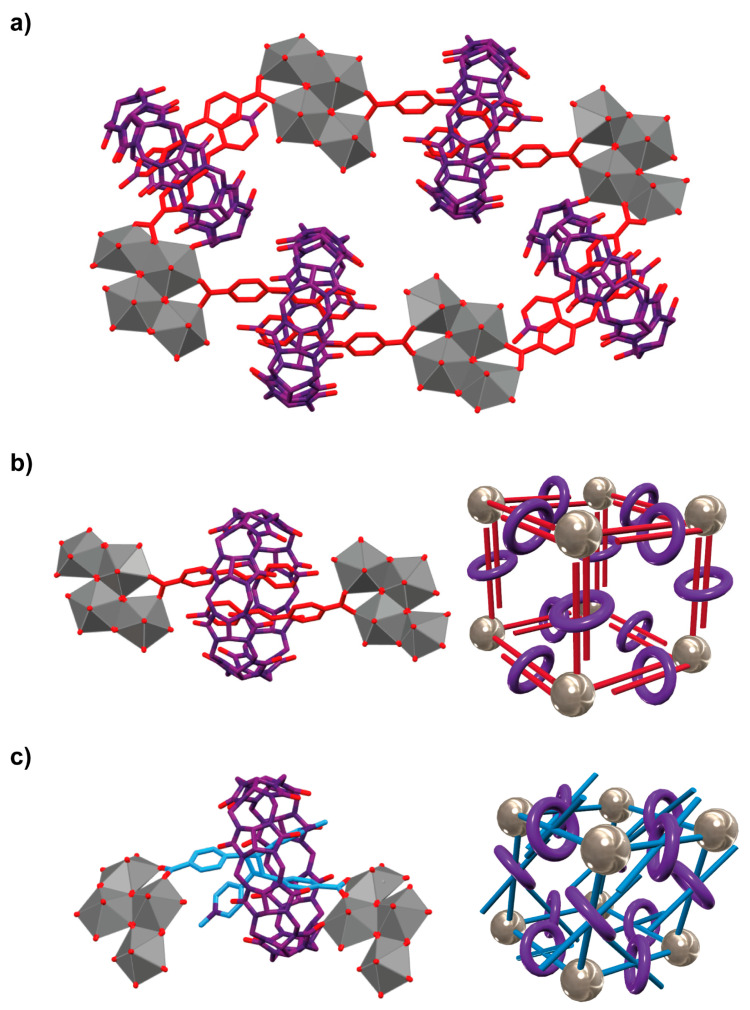
(**a**) Stick representation of the crystal structure of **U-**CB[8]-**MPyVB** showing a square grid of pseudorotaxanes connected to different uranium clusters; (**b**) stick representation of a single pseudorotaxane connected to two different uranium nodes (**left**) and cartoon representation of U-CB[8]-MPyVB (**right**); (**c**) stick representation of the crystal structure of the interlocked photodimerization product within the MOF matrix (**left**) and cartoon representation of the deformation in the crystalline array as a consequence of the photoirradiation (**right**) involving quinone loading, photorelease and thermal treatment [53]. Color key: grey = uranium atoms; purple = carbon atoms of the cucurbituril macrocycle and nitrogen atoms; red = carbon atoms of the styrylpyridinium-based compounds and oxygen atoms; blue = carbon atoms of the cyclobutane-based compounds; purple toroid = cucurbit [8]uril macrocycle; red rods = styrylpyridinium-based compounds; crossed blue rods = cyclobutane-based compounds.

Professors Mei, Feng, Shi and colleagues reported the preparation of the uranium-organic framework **U-**CB [8]-**MPyVB** by using cucurbit [8]uril-based pseudorotaxanes having a pair of carboxylic acid-functionalized styrylpyridinium linear components [53]. The synthesis of **U-CB**[8]-MPyVB proceeds via a one-pot solvothermal reaction through the assembly of uranium nitrate oxide, (*E*)-4-[2-(methylpyridine-4-yl) vinylbenzoic acid (**16**) and cucurbit [8]uril. Thus, yellow crystals of the target MOFs were obtained, in which two nonparallel styrylpyridinium guests **16** are surrounded by the cucurbituril macrocycle (Figure 18a,b). The carboxylic acid group placed at the end of each styrene-based derivative is coordinated to one different uranium cluster, preventing a dethreading process to take place. Two identical interlocked motifs, showing different conformations are established, differing in the photoactivities as a consequence of the different uranyl coordination patterns. In the photoinert motif, in which the distances between C=C bond of styrylpyridinium guests is 4.50 Å, the photodimerization is impeded. By contrast, in the photoactive motif, this distance is above 4.20 Å, fitting with Schmidt’s topochemical criteria [132], thereby allowing the photodimerization process.

Upon 365 nm irradiation of the U-CB[8]-MPyVB MOF, a single-crystal-to-single-crystal regioselective [2 + 2] photodimerization reaction of the styrene motifs in the solid state is accomplished. This reaction leads to the conversion of the interlocked styrylpyridinium linear components within the photoactive motifs into interlocked cyclobutanes (Figure 18c). Thus, a photomechanical bending of the metal-organic crystalline material is induced due to the macroscopic deformation of the MOF matrix (Figure 18b,c). Very likely, this development would allow substantial advances in the preparation of photoactuator devices with special relevance in microrobotics and optomechanics

## 9. General Remarks

The photoresponsive metal-organic frameworks highlighted in this review are highly important because of their advantageous properties and numerous potential practical applications (Table 1). This section addresses a general discussion of these MOFs, remarking some current applications and postulating some potential implementations.

Due to the intrinsic porosity that characterizes MOFs, most of them can be applied in selective adsorption or controlled cargo release (Table 1, entries 1–4 and 8) [62,63,68,70,113]. The incorporation of photoactive units in the organic struts can lead to a tailored pore size, affording suitable materials for such applications. One of the biggest challenges in this type of application is to improve the reusability of MOFs. This reusability is affected by several factors, including the partial degradation of the framework by the applied light stimulus and the persistence of part of the host molecule within the crystalline material after the release cycle. In this way, the regenerable **^DTE^MOF**, showing a reversible crystalline assembly [68], paves the way to the development of enhanced adsorption/desorption implementations, avoiding the abovementioned issues. However, this material affords an immediate release of the cargo, while other materials, such as **UMUMOF-(*E*)-3** [113], allow control of the release rate, affording a dosage of the cargo. This interlocked fumaramide-based material is limited by the photostationary state, not being able to convert most of the *trans* isomer to the *cis* one. Future research should be focused on the development of enhanced photoactive MOFs combining fast and slow release of host molecules at will.

The morphological changes induced by the application of light in these photoresponsive materials which induce different adsorption capacities or release rates may lead to consider its use in the protection of unstable reactants within the MOF matrix and its switchable release during the reaction course. In order to accomplish this interesting application, the selected wavelength should not produce any effect on the rest of reactants present in the reaction medium.

These MOFs also have potential applications in the research areas of Pharmacy and Medicine, highlighting those related to advanced drug delivery [133,134,135,136,137]. The use of photoresponsive MOFs to encapsulate drugs would permit the release at a specific time or a target place by applying a light input. The main trend of this research should be focused on the use of non-cytotoxic materials, organic struts and metal nodes. Furthermore, to accomplish biomedical applications, the selected wavelength must not damage cells or tissues. Thus, the development of photoactive metal-organic materials operating by the application of visible light turns out to be of special relevance. One potential strategy to achieve this goal could be the one followed by Professor Feringa in a molecular motor-based MOF (Table 1, entry 7), in which the incorporation of a photosensitizer strut leads to the effective phoroisomerization using green light as irradiation source through an efficient energy transfer between this photosensitizer and the photoisomerizable linker [87].

Favorable properties of these materials herald possible applications in other fields, such as Nanorobotics, Data Storage and Optoelectronics (Table 1, entries 5–7 and 9) [53,76,78,85,87].

Most of the currently implemented applications of these photoresponsive MOFs are at the research laboratory level. The spiropyran-based MOFs synthesized by Professor Shustova have been successfully used for the development of FET and LED devices (Table 1, entry 5) [76]. This technological application makes evident the importance of the incorporation of photocromic MOF scaffolds placed at a FET device in order to orthogonally control the drain current.

## 10. Summary and Outlook

The incorporation of photoresponsive molecules within MOFs has led to the preparation of materials which change their properties in response to light irradiation. In order to incorporate this photoswitchable ligands, the well-stablished synthetic protocols for obtaining this type of material have been employed, such as mixed-linker solvothermal conditions and SALE methodology. The vast design possibility forecasts materials in which future prospects are almost limitless. Additionally, the precise modulation of the photochemical stimulus, being able to modify time, wavelength and irradiation power, leads to forecasting a promising future for this field of research.

Although the use of “conventional” ligands, including azobenzenes, diarylethenes, spiropyrans and DASA, has allowed the development of a wide variety of applications, this research will still provide many results potentially allowing enhanced gas separations and preparation of new optoelectronic devices, among other functions. The combination of reticular chemistry and molecular machinery, by incorporating light-responsive molecular motors and rotaxanes inside a MOF matrix, further expands the range of options for the development of photoresponsive materials showing improved functionalities.

Despite the heartening reported results so far, this is not a fairy tale and there are still some difficulties to overcome. In addition to the issue with the photostationary equilibrium that is obtained when some of the photoresponsive ligands are used, the photochemical stimulus should fall equally on all the layers of the material. Another issue is the scale-up production of the material in order to apply these light-responsive MOFs in the industry, sometimes limited by the yield of the organic strut. Regarding industrial applications, materials having a high-performance iterability must be developed to make them cost-effective.

Photoresponsive metal-organic frameworks have a bright outlook as adjustable scaffolds to prepare smart materials, which makes this area of study really attractive to a wide range of scientists from different research fields. This interdisciplinarity would benefit the progress of these interesting porous materials.

## Figures and Tables

**Table 1:** General considerations of the selected photoresponsive MOFs [53,62,63,68,70,75,76,78,85,87,113].

Entry	MOF	Linker Type	Principal Property	Practical Implementations
**1**	**U-azo**	Azobenzene	Adsorbent	CO_2_ adsorption *
**2**	**Th-Azo-MOF**	Azobenzene	Adsorbent	Rhodamine B adsorption *
**3**	**^DTE^MOF**	Diarylethene	Reversible crystalline assembly	Highly efficient molecular dispenser of guest molecules **
**4**	**ECUT-30**	Diarylethene + azobenzene	Adsorbent	Gas capture and selective adsorption of guest molecules *
**5**	**^Spiro^MOFs**	Spiropyran	Optoelectronic	Development of FET and LED devices *
**6**	**DUT-5(indoline)_0_._5–2_._5_(DASA)**	DASA	Bistability	Development of NVM materials **
**7**	**Moto-MOFs**	Molecular motor	Unidirectional rotary motion	Photoswitchable microfluidic pumps and photodriven mass transport **
**8**	**UMUMOF-(*E*)-3**	Interlocked furamide	Adjustable porosity	Development of molecular dispensers of *p*-benzoquinone *
**9**	**U-CB**[8]**-MPyVB**	Interlocked styrylpyridinium	Regioselective photodimerization	Development of photoactuator Devices **

* Tested by the authors. ** Potential applications.

## Data Availability

Not applicable.
